# Maternal Proton Pump Inhibitor Use During Pregnancy and Risk of Low Birth Weight in Offspring in Korea, 2008-2019

**DOI:** 10.1001/jamanetworkopen.2023.7962

**Published:** 2023-04-13

**Authors:** Ahhyung Choi, Yunha Noh, Dong Keon Yon, Ju-Young Shin

**Affiliations:** 1 School of Pharmacy, Sungkyunkwan University, Suwon, South Korea; 2 Department of Biohealth Regulatory Science, Sungkyunkwan University, Suwon, South Korea; 3 Department of Epidemiology, Biostatistics, and Occupational Health, McGill University, Montreal, Quebec, Canada; 4 Center for Digital Health, Medical Science Research Institute, Kyung Hee University Medical Center, Kyung Hee University College of Medicine, Seoul, South Korea; 5 Department of Clinical Research Design & Evaluation, Samsung Advanced Institute for Health Sciences & Technology, Sungkyunkwan University, Seoul, South Korea

## Abstract

This cohort study examines whether the risk of low birth weight is associated with prenatal exposure to proton pump inhibitors.

## Introduction

Pregnant women are at increased risk of gastroesophageal reflux disease due to hormonal changes and elevated pressure on the stomach.^1^ Although lifestyle modifications are the recommended first-line therapy to treat gastroesophageal reflux disease, proton pump inhibitors (PPIs) are frequently used in unresolved symptoms.^1^ Evidence on the fetal safety of PPI use during pregnancy is limited, particularly for nonteratogenic outcomes, such as low birth weight, an important factor associated with neonatal mortality and morbidity. Preclinical studies have reported a dose-dependent risk of reduced fetal weight associated with PPI use and increased placental penetration of PPI with gestational age.^2^ However, most observational studies have focused on the teratogenic outcomes associated with PPI exposure in early pregnancy, and the association of PPI exposure during the entire pregnancy with fetal outcomes other than malformation has not been thoroughly demonstrated.^3^ To date, only 3 studies^4,5,6^ have investigated the risk of low birth weight associated with maternal PPI use, which found no association; however, with insufficient power and the sole evaluation on a specific period (eg, first trimester) and PPI (eg, omeprazole), these results are inadequate to guide clinicians and patients. We conducted a nationwide cohort study to examine the risk of low birth weight associated with prenatal PPI exposure.

## Methods

We used Korea’s National Health Insurance Service database to identify all pregnancies resulting in live births between April 1, 2008, and December 31, 2019. Pregnancies in women older than 44 years at delivery and those with missing information on perinatal birth weight were excluded. Women were considered exposed if they filled a prescription for PPIs between the start of pregnancy and the 245th day of gestation. This period was set to avoid the differential opportunity of PPI exposure between preterm and term deliveries. The reference group consisted of women without exposure to PPIs from 90 days before pregnancy to the delivery date. The outcome was low birth weight (perinatal weight <2.5 kg). To control for confounders (Table 1), we used a propensity score fine stratification–based logistic regression model to estimate the odds ratios (95% CIs). Additional details on the methods, including secondary analyses and sensitivity analyses, are in the eMethods in Supplement 1. Data were analyzed from September 1, 2022, to February 17, 2023. The study was approved by the institutional review board of Sungkyunkwan University. The requirement for obtaining informed consent was waived because this study used deidentified administrative data. This study followed the STROBE reporting guideline reporting guideline.

**Table 1.** Baseline Characteristics of the Study Cohort

Characteristic^a^	Unadjusted			Adjusted		
	Pregnancies, No. (%)		Standardized difference	Pregnancies, No. (%)		Standardized difference
	PPI exposed (n = 41 664)	Unexposed (n = 2 121 323)		PPI exposed (n = 41 649)	Unexposed (n = 2 118 941)	
Age at delivery, mean (SD), y	32.0 (4.5)	31.7 (4.1)	0.09	32.0 (4.5)	32.1 (4.4)	0.00
Age group, y						
≤20	134 (0.3)	4284 (0.2)	0.02	134 (0.3)	6457 (0.3)	0.00
21-25	2023 (4.9)	87 978 (4.1)	0.03	2021 (4.9)	100 980 (4.8)	0.00
26-30	9330 (22.4)	516 994 (24.4)	−0.05	9328 (22.4)	472 718 (22.3)	0.00
31-35	17 963 (43.1)	1 013 590 (47.8)	−0.05	17 960 (43.1)	918 975 (43.4)	−0.01
36-40	10 254 (24.6)	437 651 (20.6)	0.10	10 249 (24.6)	521 844 (24.6)	0.00
41-44	1960 (4.7)	60 826 (2.9)	0.10	1957 (4.7)	97 967 (4.6)	0.00
Medical aid recipients	444 (1.1)	9810 (0.5)	0.07	440 (1.1)	21 060 (1.0)	0.01
Income level, quartile						
First	8762 (21.0)	413 335 (19.5)	0.04	8755 (21.0)	443 343 (20.9)	0.00
Second	10 266 (24.6)	524 985 (24.7)	0.00	10 264 (24.6)	519 891 (24.5)	0.00
Third	14 084 (33.8)	747 763 (35.2)	−0.03	14 080 (33.8)	717 750 (33.9)	0.00
Fourth	8552 (20.5)	435 240 (20.5)	0.00	8550 (20.5)	437 958 (20.7)	0.00
Nulliparity	22 881 (54.9)	1 203 572 (56.7)	−0.04	22 874 (54.9)	1 167 333 (55.1)	0.00
Multiple gestation	1168 (2.8)	46 733 (2.2)	0.04	1166 (2.8)	60 473 (2.9)	0.00
Year of delivery						
2008-2011	6234 (15.0)	723 334 (34.1)	−0.46	6233 (15.0)	295 411 (13.9)	0.03
2012-2015	13 381 (32.1)	745 470 (35.1)	−0.06	13 380 (32.1)	684 813 (32.3)	0.00
2016-2019	22 049 (52.9)	652 519 (30.8)	0.46	22 036 (52.9)	1 138 717 (53.7)	−0.02
Indications						
GERD	21 326 (51.2)	107 056 (5.0)	1.20	21 312 (51.2)	1 075 514 (50.8)	0.01
Barrett esophagus	12 (0.0)	23 (0.0)	0.02	12 (0.0)	329 (0.0)	0.01
Ulcer	6342 (15.2)	73 228 (3.5)	0.41	6329 (15.2)	310 248 (14.6)	0.02
Gastritis and duodenitis	25 044 (60.1)	596 480 (28.1)	0.68	25 030 (60.1)	1 288 219 (60.8)	−0.01
Dyspepsia	6169 (14.8)	159 738 (7.5)	0.23	6159 (14.8)	308 285 (14.5)	0.01
Heartburn	1624 (3.9)	44 579 (2.1)	0.11	1621 (3.9)	81 977 (3.9)	0.00
Zollinger-Ellison syndrome	3 (0.0)	13 (0.0)	0.01	3 (0.0)	155 (0.0)	0.00
*Helicobacter pylori* infection	80 (0.2)	223 (0.0)	0.06	74 (0.2)	2816 (0.1)	0.01
Medical conditions						
Anxiety	1235 (3.0)	18 089 (0.9)	0.16	1229 (3.0)	59 220 (2.8)	0.01
Diabetes	562 (1.3)	13 377 (0.6)	0.07	561 (1.3)	28 271 (1.3)	0.00
Epilepsy	123 (0.3)	3484 (0.2)	0.03	123 (0.3)	5947 (0.3)	0.00
Headache or migraine	3900 (9.4)	101 285 (4.8)	0.18	3896 (9.4)	196 793 (9.3)	0.00
Hypertension	532 (1.3)	11 575 (0.5)	0.08	529 (1.3)	26 049 (1.2)	0.00
Renal disease	231 (0.6)	6341 (0.3)	0.04	230 (0.6)	11 679 (0.6)	0.00
Alcohol or drug dependence	66 (0.2)	1278 (0.1)	0.03	66 (0.2)	3340 (0.2)	0.00
Prescription drug use						
Antiepileptic agents	712 (1.7)	13 163 (0.6)	0.10	708 (1.7)	34 706 (1.6)	0.01
Antidepressants	2232 (5.4)	29 312 (1.4)	0.22	2225 (5.3)	105 283 (5.0)	0.02
Antidiabetic drugs	440 (1.1)	11 140 (0.5)	0.06	439 (1.1)	22 600 (1.1)	0.00
Antihypertensives	1733 (4.2)	32 869 (1.5)	0.16	1727 (4.1)	84 730 (4.0)	0.01
Benzodiazepines	10 609 (25.5)	182 629 (8.6)	0.46	10 597 (25.4)	525 564 (24.9)	0.01
Corticosteroids	20 294 (48.7)	674 244 (31.8)	0.35	20 282 (48.7)	1 042 858 (49.2)	−0.01
Fertility drugs	3143 (7.5)	162 947 (7.7)	−0.01	3142 (7.5)	162 126 (7.7)	0.00
Opioid analgesics	23 567 (56.6)	809 917 (38.2)	0.38	23 554 (56.6)	1 210 878 (57.1)	−0.01
Nonsteroidal anti-inflammatory drugs	31 048 (74.5)	1 224 058 (57.7)	0.36	31 034 (74.5)	1 598 186 (75.4)	−0.02
Thyroid hormones	2621 (6.3)	81 731 (3.9)	0.11	2620 (6.3)	135 691 (6.4)	−0.01
Obstetric comorbidity index score, mean (SD)	0.7 (1.0)	0.5 (0.8)	0.23	0.7 (1.0)	0.7 (1.0)	0.00
No. of outpatient visits, mean (SD)	8.3 (7.9)	5.3 (5.5)	0.45	8.3 (7.9)	8.3 (7.2)	0.00
No. of emergency department visits, mean (SD)	0.1 (0.5)	0.1 (0.3)	0.17	0.1 (0.5)	0.1 (0.5)	0.00
No. of hospitalizations, mean (SD)	0.1 (0.4)	0.1 (0.3)	0.12	0.1 (0.4)	0.1 (0.4)	0.00

Abbreviations: GERD, gastroesophageal reflux disease; PPI, proton pump inhibitor.

^a^ Demographic and socioeconomic characteristics, such as age, health insurance type, and income level, were assessed at delivery. Maternal medical conditions and medications used were assessed between the 6 months before pregnancy and the first trimester, and the measures of health care use were assessed during the period 6 months before pregnancy.

## Results

In the cohort of 2 162 987 pregnancies (mean [SD] maternal age, 31.7 [4.1] years), 41 664 were exposed to PPIs. After propensity score fine stratification, all baseline characteristics were well-balanced between the 2 groups (Table 1). The mean (SD) birth weights were 3.12 (0.49) kg in PPI-exposed pregnancies and 3.18 (0.46) kg in PPI-unexposed pregnancies. Compared with PPI-unexposed pregnancies, there was no increased risk of low birth weight (adjusted odds ratio, 0.95; 95% CI, 0.91-1.00) associated with PPI use during pregnancy (Table 2). The secondary analyses also revealed no increased risk of low birth weight associated with prenatal PPI use. Findings from the various sensitivity analyses remained consistent (Table 2).

**Table 2.** Association Between the Use of PPIs During Pregnancy and the Risk of Low Birth Weight

Variable	PPI-exposed group		Reference group		Odds ratio (95% CI)^a^
	No. of pregnancies	Low birth weight, No. (%)	No. of pregnancies	Low birth weight, No. (%)	
Main analysis (vs unexposed)	41 664	2357 (5.7)	2 121 323	104 056 (4.9)	0.95 (0.91-1.00)
Subgroups according to individual PPIs					
Rabeprazole	11 934	676 (5.7)	2 121 323	104 056 (4.9)	1.00 (0.92-1.08)
Esomeprazole	12 868	726 (5.6)	2 121 323	104 056 (4.9)	0.93 (0.86-1.01)
Lansoprazole	8678	482 (5.6)	2 121 323	104 056 (4.9)	0.91 (0.83-1.00)
Pantoprazole	6180	398 (6.4)	2 121 323	104 056 (4.9)	1.05 (0.95-1.16)
Omeprazole	3372	204 (6.0)	2 121 323	104 056 (4.9)	1.02 (0.88-1.17)
Subgroups according to exposure window, trimester					
First	27 575	1504 (5.5)	2 121 323	104 056 (4.9)	0.97 (0.91-1.02)
Second	8034	486 (6.0)	2 121 323	104 056 (4.9)	0.97 (0.88-1.06)
Third	8702	529 (6.1)	2 121 323	104 056 (4.9)	0.94 (0.86-1.02)
Subgroups according to cumulative dose, DDD					
<7	21 676	1180 (5.4)	2 121 323	104 056 (4.9)	0.95 (0.90-1.01)
7 to <14	9771	526 (5.4)	2 121 323	104 056 (4.9)	0.92 (0.85-1.01)
≥14	10 217	651 (6.4)	2 121 323	104 056 (4.9)	1.02 (0.94-1.11)
Sensitivity analysis					
≥2 PPI prescriptions	9446	591 (6.3)	2 121 323	104 056 (4.9)	1.01 (0.93-1.10)
Compared with H_2_RA	35 418	1823 (5.1)	94 578	5023 (5.3)	0.99 (0.94-1.05)
Restrict to first-time pregnancy	22 881	1448 (6.3)	1203 572	67 629 (5.6)	0.95 (0.90-1.01)
Restrict to singleton pregnancy	40 496	1722 (4.3)	2074 590	74 982 (3.6)	1.03 (0.98-1.08)
Adjust for BMI and smoking status	23 827	1324 (5.6)	1085 333	54 142 (5.0)	0.95 (0.88-1.01)
Restrict to maternal age <35 y	29 450	1495 (5.1)	1622 846	72 513 (4.5)	0.97 (0.92-1.03)
Sibling design	13 678	522 (3.8)	754 382	27 854 (3.7)	0.87 (0.76-1.00)

Abbreviations: BMI, body mass index; DDD, defined daily dose; H_2_RA, histamine_2_ receptor antagonists; PPI, proton pump inhibitor.

^a^ Propensity score–weighted odds ratio.

## Discussion

This cohort study indicated that the use of PPIs any time during pregnancy was not associated with an increased risk of low birth weight. These results are in line with previous studies^4,5,6^ that found no associations between prenatal PPI exposure and low birth weight. The large cohort in this study allowed us to evaluate risk by individual PPIs, trimester, and cumulative dose, which all showed no associations.

Study limitations include residual confounding and the possibility of misclassification of the pregnancy period. In addition, ascertainment of PPI exposure was based on prescription data, which may have led to misclassification. However, redefining the exposure as 2 or more prescriptions in sensitivity analyses did not change our results. Overall, our findings provide reassurance on the fetal safety of PPI use while enlarging the available epidemiological body of evidence.

## Notes

Supplement 1. **eMethods.** Supplemental Methods

Supplement 2.
Data Sharing Statement
