# Supplementary material for: Maternal Proton Pump Inhibitor Use During Pregnancy and Risk of Low Birth Weight in Offspring in Korea, 2008-2019
Source: JAMA Netw Open. 2023 Apr 13;6(4):e237962. doi: 10.1001/jamanetworkopen.2023.7962 (PMC10102871; doi:10.1001/jamanetworkopen.2023.7962)
Supplement: Supplement 1. — eMethods. Supplemental Methods [file jamanetwopen-e237962-s001.pdf]

# Supplementary Online Content

Choi A, Noh Y, Yon DK, Shin JY. Maternal proton pump inhibitor use during pregnancy and risk of low birth weight in offspring in Korea, 2008-2019. *JAMA Netw Open*. 2023;6(4):e237962. doi:10.1001/jamanetworkopen.2023.7962

**eMethods.** Supplemental Methods

This supplementary material has been provided by the authors to give readers additional information about their work.

**eMethods.** Supplemental Methods

***Data source and study cohort***

We conducted a retrospective cohort study using the National Health Insurance Service (NHIS) database (2008-2019), which covers the entire population of South Korea. The mother-child linked database that we used is internally constructed by the NHIS based on the unique insurance identification number shared by the family members. Using this database, we identified all pregnancies resulting in live births between April 1, 2008, and December 31, 2019. Pregnancies in women aged >44 years at delivery and those with missing information on perinatal birth weight were excluded. The gestational age was estimated based on the delivery date ascertained in the NHIS mother-child linked database and diagnostic information on preterm birth, using a previously validated algorithm in administrative healthcare database[1].

***Secondary analyses and sensitivity analyses***

As a secondary analysis, we examined the association by the timing of PPI exposure (first, second, and third trimester, ending in 245$^{th}$ day of gestation), individual PPI medications, and cumulative dose. Sensitivity analyses included redefining the exposure as ≥2 PPI prescriptions, comparison with pregnancies exposed to histamine 2 receptor antagonists, restriction to first-time pregnancies, restriction to singleton pregnancies, restriction to those available with BMI and smoking status data, excluding pregnancies with maternal age >35 years at delivery, and sibling design.

***Statistical analysis***

Baseline characteristics were compared between PPI-exposed and unexposed pregnancies using standardized mean differences; a value less than 0.1 indicated a balance in characteristics between the 2 groups. To control for potential confounders listed in Table 1, we used a propensity score (PS) fine stratification-based logistic regression model to estimate the odds ratios (ORs) with 95% confidence intervals (CIs)[2]. All statistical analyses were performed using SAS Enterprise version 7.1 (SAS Institute Inc).

**eReferences**

1. Margulis, Andrea V., et al. "Algorithms to estimate the beginning of pregnancy in administrative databases." Pharmacoepidemiology and drug safety 22.1 (2013): 16-24
2. Desai RJ, Rothman KJ, Bateman BT, Hernandez-Diaz S, Huybrechts KFJE. A Propensity score based fine stratification approach for confounding adjustment when exposure is infrequent. 2017;28(2):249.

© 2023 Choi A et al. *JAMA Network Open.*
