# Supplementary material for: Maternal Proton Pump Inhibitor Use During Pregnancy and Risk of Low Birth Weight in Offspring in Korea, 2008-2019
Source: JAMA Netw Open. 2023 Apr 13;6(4):e237962. doi: 10.1001/jamanetworkopen.2023.7962 (PMC10102871; doi:10.1001/jamanetworkopen.2023.7962)
Supplement: Supplement 2. — Data Sharing Statement [file jamanetwopen-e237962-s002.pdf]

Data Sharing Statement

Choi. Maternal Proton Pump Inhibitor Use During Pregnancy and Risk of Low Birth Weight in Offspring in Korea, 2008-2019. *JAMA Netw Open*. Published April 13, 2023. doi:10.1001/jamanetworkopen.2023.7962

Data
Data available: No
